# Editorial: Special issue: “Impact of lifestyle und behavioral risk factors on endothelial function and vascular biology”—how lifestyle and behavioral risk factors affect the vasculature

**DOI:** 10.1007/s00424-023-02826-8

**Published:** 2023-06-04

**Authors:** Andreas Daiber, Omar Hahad, Thomas Münzel

**Affiliations:** 1 Department of Cardiology, Cardiology I, University Medical Center Mainz, Mainz, Germany; 2 German Center for Cardiovascular Research (DZHK), Partner Site Rhine-Main, Mainz, Germany

The global burden of disease (GBD) has shifted from communicable, maternal, perinatal, and nutritional causes to non-communicable diseases such as atherosclerosis or metabolic disease within the last 20–30 years [1, 2]. In 2010, the leading risk factors and diseases responsible for global deaths were hypertension, ischemic heart disease, smoking, and cerebrovascular disease, accounting for 55% of the global mortality [1, 2]. In addition, genetic (familial) predisposition for non-communicable chronic diseases seems to be outcompeted by environmental risk factors, reflected by the notion that the genetic background loads the gun, but the environment pulls the trigger [3, 4]. This postulate is supported by statistics on cardiovascular and cancer-associated deaths, where only 0.25 (16.4%) of 1.53 million deaths in Western Europe in 2000 could be attributed to genetic causes [5]. Environmental science experts postulate that approximately two-thirds of all non-communicable diseases and related deaths are caused by harmful environmental exposures, and only one-third is due to genetic predisposition [6, 7]. To address this new paradigm, a new research field was defined by the term “exposome” [8], which comprises the changes of our endogenous biochemical systems by life-long environmental, behavioral, and lifestyle exposures and our social environment as well as the associated health effects [6, 9].

According to reports of the Lancet Commission on Pollution and Health [10], the World Health Organization [11], and the Global Burden of Disease Study [12, 13], environmental risk factors significantly contribute to the global burden of disease. All forms of pollution together were responsible for 9 to 12.6 million deaths in 2015 and 2012 [10, 11], respectively, reflecting 16–20% of total mortality worldwide and representing a higher number of annual deaths than estimated for smoking. Based on the most recent studies, 8.8 million deaths are attributable to ambient air pollution alone [14, 15]. These estimates do not include excess deaths caused by the non-chemical environmental health risk factors such as mental stress, noise, light exposure, and climatic change [7, 16]. There is also increasing evidence that lifestyle and behavioral risk factors in the environment, such as smoking and sedentary lifestyle, may facilitate the development of chronic non-communicable diseases of cardiometabolic origin. Tobacco smoking is a significant trigger of chronic non-communicable disease and a risk factor for cardiovascular and lung disease, causing 8.7 million global deaths (15.4% of all deaths) [17]. Whereas global tobacco use was reduced during the last two decades, mostly due to a reduction of female smokers, the use of E-cigarettes, novel nicotine delivery systems such as heat-not-burn products and waterpipes (shisha), shows a pandemic growth, mainly due to flavoring of tobaccos and liquids, a lower starting age of users, facilitated usage, or higher nicotine content causing faster addiction [18]. Likewise, alcohol abuse is another lifestyle drug-dependent cardiovascular health issue [19]. Western societies and emerging market countries also face an increasing rate of people with sedentary lifestyles or over-nutrition (reflected by high body mass index), risk factors that are well-known to trigger cardiometabolic disease by impairment of beneficial vascular function [20, 21]. Figure 1 provides a summary and comparison of the leading lifestyle health risk factors and puts them into relation with known cardiometabolic sequelae such as high fasting blood glucose and high- and low-density lipoprotein (LDL) cholesterol, as well as the leading health risk factor, high systolic blood pressure that was responsible for 10.8 million global deaths (19.2% of all deaths) in 2019 [17].

**Fig. 1:**
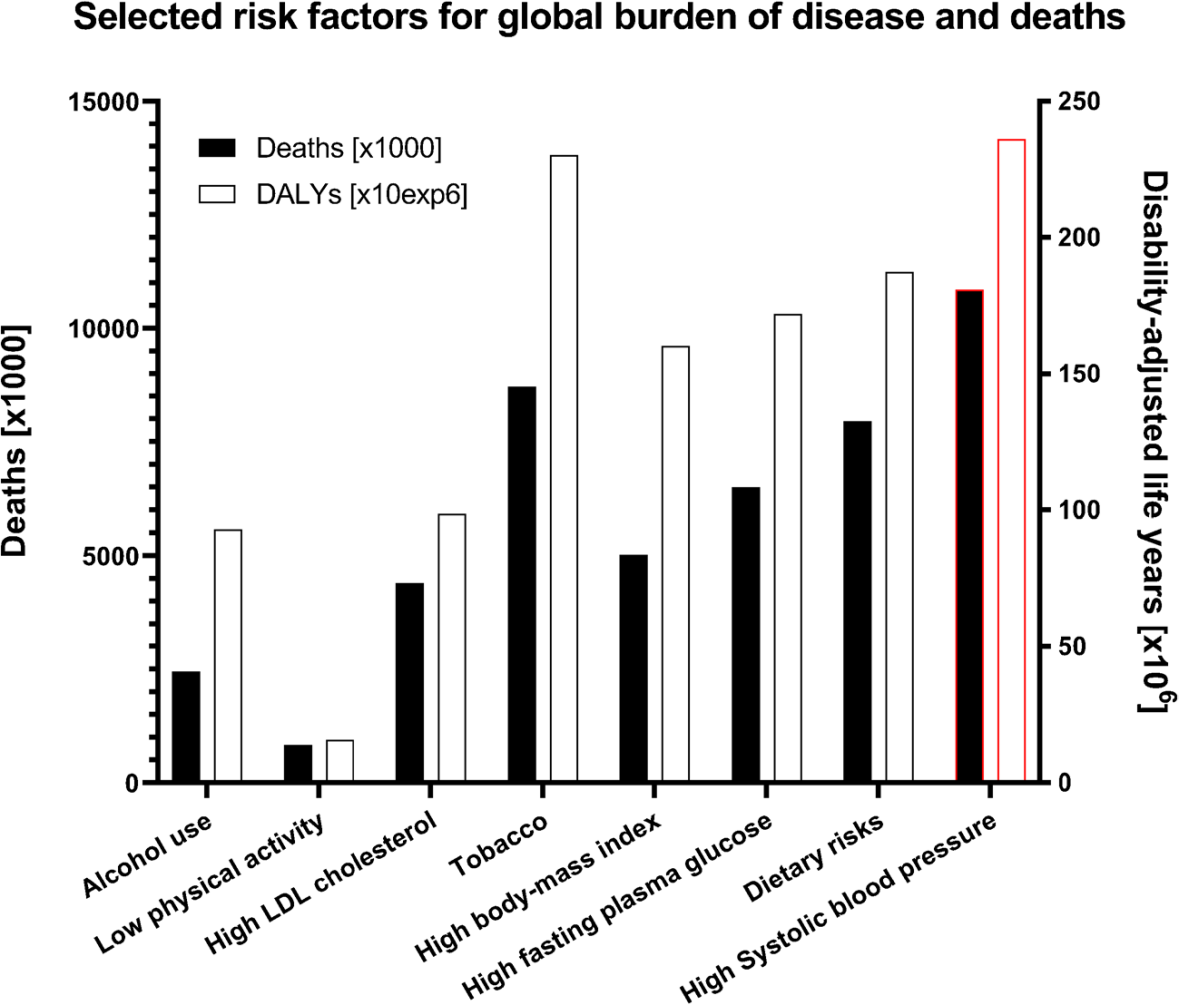
Selected risk factors for the global burden of disease (disability-adjusted life years (DALYs)) and deaths presented as the sum for female and male population. High systolic blood pressure was added as the benchmark and leading classical health risk factor (marked by red color). Lifestyle risk factors, alcohol use, low physical activity, tobacco smoking, and high body mass index/dietary risks (e.g., over-nutrition) represent leading health risk factors related to the specific (individual) exposome. High- and low-density lipoprotein (LDL) cholesterol, high fasting plasma glucose, and high systolic blood pressure may represent direct consequences of the specific exposures, the lifestyle. Data are based on the results of the Global Burden of Disease Study [17]. Tabular data used with permission under the Creative Commons Attribution (CC BY 4.0)

The present special issue provides an overview of the health effects of tobacco cigarette and shisha smoking, E-cigarette vaping, usage of other novel nicotine delivery systems, sedentary lifestyle or over-nutrition, and alcohol use with a focus on adverse effects on endothelial function and cardiovascular health. The negative effects of the toxic constituents of these tobacco (replacement) products, as well as the beneficial effects of exercise or fasting on different biological pathways, will also be discussed, especially regarding their impact on key vascular processes such as nitric oxide signaling, smooth muscle proliferation, remodeling, and atherosclerosis, in part driven by oxidative stress and inflammatory cascades. With the present Special Issue, we want to highlight the pathomechanisms underlying cardiovascular and metabolic disorders in response to environmental (especially lifestyle) risk factors and the existing research gaps. We will also emphasize emerging mechanisms based on dysregulation of epigenetic pathways by environmental lifestyle and behavioral risk factors.

## Smoking, vaping, and other tobacco replacement strategies

The overview provided by Daiber et al. highlights the threats and benefits of E-cigarette vaping in comparison with classical tobacco cigarette and shisha smoking [22]. The toxic compounds comprised in smoke and vapor are discussed in detail, also with respect to their potential harmful effects on endothelial function, oxidative stress, inflammation, and damage of the cardiovascular system. Also evidence for pathomechanistic overlap between smoking and vaping regarding endothelial dysfunction and cardiometabolic disease is provided. The review by Hahad et al. emphasizes negative effects of smoking on vascular biology from a human perspective [23]. Highlights are a summary of human field studies on impairment of endothelial function by tobacco smoking, mitigation of these negative side effects by administration of antioxidants, and proposed pathomechanistic events centered around oxidative stress and inflammation. In line with these review articles, a cell culture study by Kuntic et al. demonstrates that acrolein, a major toxic aldehyde in E-cigarette vapor, can induce oxidative stress in cultured endothelial cells and macrophages [24]. Focus of this experimental study is related to determination of intracellular and extracellular formation of reactive oxygen species by different methods, cell viability, and activation of the phagocytic NADPH oxidase. Giebe et al. contribute a complementary cell culture study on the induction of inflammation by cigarette smoke versus next-generation tobacco and nicotine product extracts in cultured human monocytes [25]. Besides antioxidant response genes (e.g., Nrf2-related) and Nox2 as a source of oxidative stress, a battery of inflammatory biomarkers such as interleukins and tumor necrosis factor was assessed. In line with these experimental data, the review by Klein et al. focuses on the pathophysiological effects of cigarette smoke and next-generation tobacco and nicotine products on endothelial function [26]. Insights into pathomechanistic details related to inflammation, oxidative stress, and oxidative damage of endothelial nitric oxide synthase are provided with an outlook on the functional and clinical consequences.

## Sedentary lifestyle, alcohol abuse, and unhealthy nutrition versus exercise and fasting

Fasipe et al. provide a review on the cardiometabolic health risks of sedentary lifestyle and the beneficial effects of physical exercise [27]. The authors focus on oxidative stress as a pathomechanism of sedentary lifestyle, discuss the sources of reactive oxygen species and the resulting oxidative damage in detail, also for the different involved cell types such as endothelial, smooth muscle and adipose tissue cells. They also highlight how exercise can mitigate these pathophysiological processes, focusing on NRF2 as a central mediator of protection. Dikalov et al. contributes a review on the adverse health effects of cigarette smoking and dietary as well as sedentary lifestyle risks [28]. Detailed pathomechanisms for all three health risk factors are discussed, including telomerase expression/activity impairment, sirtuin-3 depletion and inactivation, all of which contributes or is related to enhanced oxidative stress, mitochondrial dysfunction and subsequent endothelial dysfunction and hypertension as well as associated cardiometabolic sequelae. Finally, future pharmacological strategies but also caloric restriction for intervention are presented. The review by Li and Xia reports on the so-called love-hate relationship for alcohol consumption and cardiovascular health and disease [29]. Accordingly, the beneficial effects of low-to-moderate alcohol intake are discussed and contrasted to the clearly harmful effects of alcohol abuse. The authors present numerous human studies for the pros and cons of alcohol usage, discuss the pathomechanisms, and highlight the processes underlying the beneficial effects, both based on redox regulatory effects. The overview by Ricci et al. deals with maternal nutrition effects on vascular function in the offspring [30]. The impact of not only maternal high fat diet but also energy and protein restriction is discussed in detail with deep mechanistic insight on the effects on the vascular system, especially the endothelial nitric oxide synthase and tetrahydrobiopterin pathways. Oxidative stress and inflammation are identified as major pathomechanisms for malnutrition. The work by Man et al. reviews the hypertensive disorders and vascular dysfunction of pregnancy (e.g., preeclampsia) and how cardiovascular risk can be propagated to the offspring, also focusing on fetal programming and reprogramming [31]. The authors discuss in detail how dietary supplements such as citrulline, arginine, vitamins, and resveratrol can interfere with the adverse effects of pregnancy cardiometabolic disorders on the offspring.

## Concluding note

Lifestyle risk factors share common pathophysiological pathways centered on oxidative stress and inflammation. Oxidative stress and inflammation also represent hallmarks of all cardiovascular, neurodegenerative, and metabolic diseases. Due to this overlap of these central pathomechanisms, we may expect additive/synergistic adverse biochemical effects in response to environmental, lifestyle, and traditional health risk factors leading to aggravated pathogenesis of non-communicable diseases in a bonfire fashion [32, 33]. Whereas the general exposome (e.g., exposures such as air pollution, climate change, traffic noise, lack of green space) cannot be easily modified by the individual, the specific exposome defined by behavioral and lifestyle risk factors can be largely changed individually. Accordingly, lifestyle changes can significantly impact the global disease burden and population health, thereby representing an attractive possibility to decrease health costs and socioeconomic burdens caused by chronic non-communicable diseases. With this, we would like to thank all the authors for their valuable contributions to this special issue, and we hope that they will stimulate new ideas and scientific discussion.
